# High-Throughput Phenotyping of Fire Blight Disease Symptoms Using Sensing Techniques in Apple

**DOI:** 10.3389/fpls.2019.00576

**Published:** 2019-05-10

**Authors:** Sanaz Jarolmasjed, Sindhuja Sankaran, Afef Marzougui, Sarah Kostick, Yongsheng Si, Juan José Quirós Vargas, Kate Evans

**Affiliations:** ^1^Department of Biological Systems Engineering, Washington State University, Pullman, WA, United States; ^2^Donald Danforth Plant Science Center, St. Louis, MO, United States; ^3^Tree Fruit Research and Extension Center, Washington State University, Wenatchee, WA, United States; ^4^College of Information Science and Technology, Agricultural University of Hebei, Baoding, China

**Keywords:** proximal sensing, unmanned aerial systems, normalized difference spectral indices, classification, *Malus pumila* Mill

## Abstract

Washington State produces about 70% of total fresh market apples in the United States. One of the primary goals of apple breeding programs is the development of new cultivars resistant to devastating diseases such as fire blight. The overall objective of this study was to investigate high-throughput phenotyping techniques to evaluate fire blight disease symptoms in apple trees. In this regard, normalized stomatal conductance data acquired using a portable photosynthetic system, image data collected using RGB and multispectral cameras, and visible-near infrared spectral reflectance acquired using a hyperspectral sensing system, were independently evaluated to estimate the progression of fire blight infection in young apple trees. Sensors with ranging complexity – from simple RGB to multispectral imaging to hyperspectral system – were evaluated to select the most accurate technique for the assessment of fire blight disease symptoms. The proximal multispectral images and visible-near infrared spectral reflectance data were collected in two field seasons (2016, 2017); while, proximal side-view RGB images and multispectral images using unmanned aerial systems were collected in 2017. The normalized stomatal conductance data was correlated with disease severity rating (*r* = 0.51, *P* < 0.05). The features extracted from RGB images (e.g., maximum length of senesced leaves, area of senesced leaves, ratio between senesced and healthy leaf area) and multispectral images (e.g., vegetation indices) also demonstrated potential in evaluation of disease rating (|*r*| > 0.35, *P* < 0.05). The average classification accuracy achieved using visible-near infrared spectral reflectance data during the classification of susceptible from symptomless groups ranged between 71 and 93% using partial least square regression and quadratic support vector machine. In addition, fire blight disease ratings were compared with normalized difference spectral indices (NDSIs) that were generated from visible-near infrared reflectance spectra. The selected spectral bands in the range 710–2,340 nm used for computing NDSIs showed consistently higher correlation with disease severity rating than data acquired from RGB and multispectral imaging sensors across multiple seasons. In summary, these specific spectral bands can be used for evaluating fire blight disease severity in apple breeding programs and potentially as early fire blight disease detection tool to assist in production systems.

## Introduction

Apple (*Malus pumila* Mill) belongs to the *Rosaceae* family, and is the most consumed and valued fruit crop in the United States (Lu and Lu, 2017) and other parts of the world. The United States is the second largest apple producer worldwide and Washington State has the nation’s top apple production area. Washington State’s favorable climate with low humidity assists in the control of many of the typical apple diseases (Sutton, 1996). However, the introduction of new cultivars with different levels of disease susceptibility has revealed that in some growing seasons, the bacterial disease fire blight can be a problem. Fire blight is a major concern to commercial fruit production, as it results in significant production losses (Sutton, 1996; Salm and Geider, 2004). The causative agent of fire blight, *Erwinia amylovora* (Bereswill et al., 1995) can infect flowers, fruits, shoots, and the rootstock of the tree, potentially causing flower, tissue, and/or tree death (Norelli et al., 2003). *E. amylovora* uses wounds or natural openings as well as nectarthodes to enter the host (Vanneste, 2000).

Blackened crooked shoots (i.e., shepherd’s crook), bacterial ooze, necrotic leaves, and the formation of necrotic lesions and cankers are symptoms that characterize typical fire blight shoot infections (Van der Zwet et al., 2012). Fire blight infections can result in structural damage and potentially tree death. Several factors impact the incidence and severity of fire blight symptoms such as environmental conditions, cultivar susceptibility, host vigor, amount of inoculum present, and management practices (Van der Zwet et al., 2012). Most modern commercial apple cultivars are susceptible to fire blight and current control methods (e.g., pruning, antibiotics) are not effective against all disease phases (e.g., floral, shoot, rootstock) and/or are not sustainable. One major limitation of disease control is the ability of pathogens to survive for a long period of time, becoming active when favorable weather conditions prevail. The use of antibiotics for control is limited by both legislation and to prevent the development of resistant strains (Lespinasse and Aldwinckle, 2000). Breeding new cultivars of apple that are resistant to fire blight is a logical progression to solve this issue (Kostick et al., 2019). The Washington State University (WSU) apple breeding program focuses on producing long storing, high quality cultivars developed for local production and has recently added resistance to fire blight as an important selection trait (Harshman et al., 2017).

Phenotyping fire blight susceptibility is challenging due to the large impacts of environment and host growth status on susceptibility, quantitative resistance, and the inconsistent nature of fire blight (Brown, 2012). Fire blight susceptibility to shoot infection has typically been evaluated by measuring the current season’s shoot length and the lesion (necrosis) or healthy tissue length. The proportion of healthy or necrotic tissue can be calculated, which provides a standardized measure of infection (Durel et al., 2009). As such visual evaluations are labor intensive and subjective; the development of an accurate and rapid sensing technique for high-throughput screening of fire blight symptoms in apples would be beneficial.

In recent years, high-throughput phenotyping using non-invasive imaging and sensing systems has made advances toward evaluation of anatomical, physiological, and biochemical properties in plants (Mahlein, 2016). High-throughput phenotyping is currently being developed for grain crops such as wheat (Casadesús et al., 2007; Hosoi and Omasa, 2009; Römer et al., 2011), corn (Trachsel et al., 2011; Weber et al., 2012; Yang et al., 2014), and rice (Tanabata et al., 2012; Hairmansis et al., 2014). In spite of the progress in sensing systems, there are limited studies on optical spectrometric and imaging techniques for phenotyping diseases in trees. Current studies in sensor-based disease detection are focused on identifying onset of disease for management applications (Bock et al., 2008; Wijekoon et al., 2008; Neumann et al., 2014). Meanwhile, in breeding programs, the focus has been to quantify the extent of disease (resistant/susceptible) under inoculated conditions, excluding the genotypic differences in morphology.

In a controlled environment study (Delalieux et al., 2007), hyperspectral reflectance spectra were used to detect apple scab disease in tree leaves. Susceptible and resistant apple cultivars were inoculated with conidia of *Venturia inaequalis*. The study indicated that spectral reflectance between 1,350–1,750 nm and 2,200–2,500 nm were effective in distinguishing between healthy and infected leaves. The area under the receiver operator characteristic plots, indicated as *c*-value, was used as a measure of the discriminatory performance. A good predictability in classification of infected and healthy trees using logistic regression, partial least squares logistic discriminant analysis, and tree-based modeling (*c*-value > 0.8) was achieved. Similarly, Bauriegel et al. (2014) assessed the disease rating (caused by a fungal pathogen infection, *Bremia lactucae*) on 10 Butterhead and Batavia lettuce cultivars in a semi-controlled environment. Leaves were inoculated by spraying a conidia suspension and cultivars were rated visually for 14 days. A chlorophyll fluorescence imaging system was used to capture images each day after dark adaptation of leaves for 10 min from leaf discs. The ratio of variable fluorescence to maximum fluorescence was calculated for each leaf disc. In pixel-wise analysis of images, in 10–14 days after inoculation, a significant decrease in the ratio up to 0.35 in susceptible cultivars was observed, while the ratio was 0.10 for resistant cultivars. The potential for rapid and automated cultivar resistance detection using their sensing system was reported. Díaz-Varela et al. (2015) used unmanned aerial vehicle (UAV)-based imaging to estimate olive tree height and crown diameter for breeding applications. The results showed 3–16% of root mean square error between the sensing data estimates and field measurements. Other studies in literature (Gomez-Candon et al., 2014; Virlet et al., 2014) report the application of multispectral imaging (RGB, near infrared, and thermal infrared) to assess apple tree response to drought.

Diverse studies demonstrated the potential of sensing techniques for disease detection in both controlled environment and field conditions for precision agriculture applications. Khanal et al. (2017) reviewed the application of thermal sensing for stress monitoring and Dlamini et al. (2019) described numerous remote sensing indices that are relevant for disease mapping. Therefore, remote sensing techniques are widely employed in monitoring and managing crops (Usha and Singh, 2013). For instance, Salgadoe et al. (2018) found that the simplified ratio vegetation index (SRVI) extracted from the high resolution Worldview-3 satellite imagery was strongly correlated with four levels of disease severity resulting from Phytophthora root rot in avocado trees. Similarly, Zarco-Tejada et al. (2018) evaluated the performance of spectral signatures derived from airborne imaging spectroscopy and thermography in detecting the early stage of *Xylella fastidiosa* infection in olive trees. In addition, Al-Saddik et al. (2018) used red–green–blue and hyperspectral imaging to detect two grapevine diseases: Yellowness and Esca. In their study, texture and spectral features extracted from imaging data were able to classify healthy and infected grapevine leaves. Furthermore, the complex data acquired using these sensing techniques required advanced tools for data processing and analysis. Tzionas et al. (2005) extracted morphological features to classify leaves through image processing integrated feed forward neural network approaches. Machine learning methods improve disease detection accuracies, especially if the methods are integrated with hyperspectral data (Golhani et al., 2018) in a 3D environment (Roscher et al., 2016). Other studies reporting the use of machine learning and/or deep learning methods for disease detection/classification can be found in literature (Meunkaewjinda et al., 2008; Phadikar and Sil, 2008; Weizheng et al., 2008).

In this research, multiple sensing techniques at different scales (proximal and remote) were evaluated to assess the fire blight infection levels in different apple cultivars, important breeding parents, and seedlings. Prior to utilizing technology to perform sensor-based high-throughput phenotyping to assess disease severity in a specific crop (such as fire blight resistance in apple), it is important to evaluate and understand the benefits and limitations of each technique. Such evaluations are more challenging in field conditions as multiple season data is required for confident evaluation of sensors and assess cultivar response to stress conditions. In this regard, the current study contributes to the application of high-throughput sensor-based assessment of fire blight disease symptoms using several proximal and remote sensing technologies for high-throughput phenotyping in apple under field conditions. Such studies on fire blight symptoms or other disease evaluation in apple breeding program has not been reported. The studies on disease detection for precision agricultural applications (e.g., crop management) may not be applicable in breeding programs, as the studies often focus on one or few varieties and early detection of diseases; while, 10s–100s of varieties are assessed for a scale in disease severity in breeding studies. Therefore, three major sensing approaches at varying complexity that were independently evaluated in this study were: (1) side-view RGB imaging to detect necrosis extent in multiple cultivars; (2) top-view remote sensing using multispectral imaging at different scales; and (3) proximal hyperspectral sensing (350–2,500 nm). In addition to disease rating by manual methods, stomatal conductance was measured to understand the physiological changes in the canopy upon infection.

## Materials and Methods

### Plant Materials and Inoculation

Data collection was performed on a set of 72 unique apple individual trees (e.g., cultivars, important breeding parents, seedlings). The trees were part of a larger planting, located at the WSU Columbia View Orchard, Douglas County, WA, United States (47°33′52.1″N, 120°14′47.3″W). They were budded onto M.111 rootstocks and planted with 82 cm spacing between trees. Full details of the germplasm, orchard establishment and maintenance can be found in Kostick et al. (2019). Freeze-dried *E. amylovora* 153n was used for inoculation and inoculum was diluted using 0.05 M dibasic phosphate buffer, pH 7 to a concentration of 5 × 10^8^ CFU mL^-1^ in 2016 and 1 × 10^9^ CFU mL^-1^ in 2017. Three to ten independent, actively growing shoots with ideally ≥15 cm of growth were chosen per tree for inoculation on April 28–29, 2016 and May 18, 2017. In 2017, 68 trees were evaluated as four trees died. Kostick et al. (2019) describes the inoculation procedure in detail.

Data collection using multiple sensors (Supplementary Figure S1 and Supplementary Table S1) was performed as described in detail in the following sessions. Most of the sensor data collection was performed in complete-disease developmental stages, which corresponded to about 40 days after inoculation (DAI; 9 June 2016 and 27 June 2017). The hyperspectral reflectance data was also collected at mid-disease development stages at about 23 DAI (19 May 2016 and 13 June 2017). Visible development of disease symptoms ceased at about 40 DAI and the disease symptoms were manually/visually rated as described in the following session at that time.

### Disease Severity Rating and Stomatal Conductance

Several disease severity rating systems are used to quantify the severity of fire blight infection (Van Der Zwet and Keil, 1979). These ratings are based on proportion of current season’s growth that was blighted or healthy, percentage of shoots that were infected per tree (i.e., incidence), and the age of wood that a lesion progressed into (i.e., infected). As ground reference data (both years), the length of current season’s shoot growth was measured at inoculation in 2016 or at the time of lesion measurement in 2017. As described by Kostick et al. (2019), disease progression within the current season’s shoot growth was evaluated by measuring the length of non-necrotic (i.e., healthy) tissue in 2016 or length of necrotic fire blight lesions in 2017. From these measurements, the proportion of healthy tissue was calculated on a given shoot and was averaged for each tree. These disease severity ratings are described as the proportion of shoot length blighted henceforth. Each shoot was also rated based on the age of wood that the lesion extended into with 0, 1, 2, and 3 representing no infection, first year, second year, and third year wood, respectively. When a lesion extended into the previous season’s wood or into third year wood, the response was considered highly susceptible. In symptomless responses, no lesions were visible and only minor responses were observed on the inoculated leaves. In 2016, average disease severity rating for each tree was used as ground reference data; while in 2017, the individual shoots that were used for hyperspectral data collection were used as ground reference data during hyperspectral data analysis, in addition to average disease severity rating for individual tree during RGB and multispectral image data analysis.

In 2017, a portable photosynthesis system (LI-6800, LI-COR Biosciences, Lincoln, NE, United States) was used to collect stomatal conductance data at the complete disease development stage (about 40 DAI) from 20 trees (subset of 72). The rationale behind the use of this system was to evaluate whether the physiological measurements such as stomatal conductance could be used as alternative to disease severity rating. Data were collected from three healthy and three inoculated leaf samples from each of the 20 trees. The stomatal conductance data were normalized by subtracting the inoculated leaves from healthy leaves in each cultivar to eliminate the cultivar effect on stomatal conductance values. The stomatal conductance of healthy leaves ranged between 186 and 473 mmol.m^-2^.s^-1^.

### RGB Image Acquisition, Image Processing, and Feature Extraction

In regard to sensor data, the sensors with ranging complexity – from simple RGB to multispectral imaging to hyperspectral system –were independently evaluated. This is important for practical application in disease symptoms evaluation as the sensor should be easy to use and data processing should be simple for breeders to adopt the technology for high-throughput phenotyping. The rationale behind the use of RGB imaging system to evaluate disease symptoms was that the disease rating was directly associated with visible symptoms and measure of length of necrotic fire blight lesions, which could be captured using RGB imaging. In regard to the use of multispectral imaging at different scales (proximal and remote) for disease resistance evaluation, it was hypothesized that although limited shoots were inoculated within the tree canopy, the presence of fire blight pathogen may induce overall canopy stress response that can be captured using generic vegetation indices extracted from multispectral images. The rationale behind the use of hyperspectral system was to capture the entire spectral reflectance response of the leaves to the disease, so as to derive novel spectral indices that can be translated into customized multispectral imaging sensors that can be integrated with ground or aerial platforms in future. The details on multispectral and hyperspectral data collection and analysis is described in the following sessions.

**FIGURE 1:**
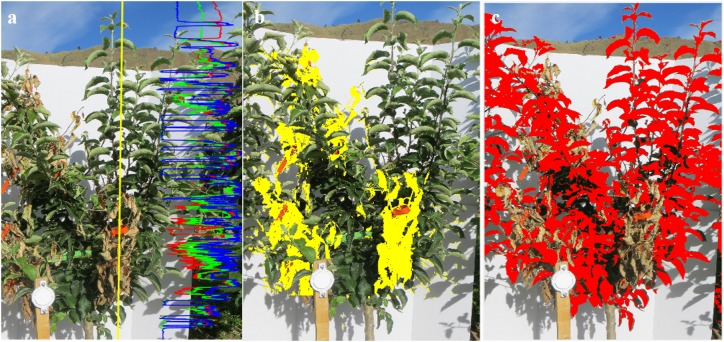
**(a)** Original RGB side-image with red, green, and blue lines in right side showing the relative gray scale intensities in the R, G, and B channels, respectively, of the yellow line highlighted in the image; **(b)** processed RGB image where the senesced leaves identified during image processing are marked in yellow; and **(c)** processed RGB image where the healthy leaves identified during image processing are marked in red. The total number of pixels representing senesced and healthy leaves, and maximum length of senesced leaf area were used for feature extraction.

An RGB digital camera (Canon PowerShot SX260HS, Carlstadt, NJ, United States) with resolution of 4000 × 3000 was used to capture side-view images of the trees with a white background board placed behind each tree (in 2017). The reference panel was placed at the tree trunk and the distance between the camera and the tree was maintained at around 1.5 m. During image processing, the first step was to observe image reflectance value patterns (Figure 1a) in each band across green leaves, senesced leaves, and shoots in Matlab^®^. This was important to separate the abaxial leaf surfaces from partially senesced leaves. Upon optimization of image processing protocol, wilted and necrotic leaves could be separated from healthy leaves using defined red, green, and blue channel reflectance values (Eq. 1).

(1) $$\begin{array}{c} I_{\left(i,j\right)}=\text{Infected pixels}\\ 60>\left(R_{\left(i,j\right)}-G_{\left(i,j\right)}\right)\geq 0\text{ and }(G_{\left(i,j\right)}-B_{\left(i,j\right)})\\ I_{\left(i,j\right)}=\text{background pixels OTHERWISE} \end{array}>0$$

where: *R*_(*i,j*)_, *G*_(*i,j*)_ and *B*_(*i,j*)_ are the red, green, and blue pixel intensities (0–255), respectively, and (*i,j*) represents each pixel coordinates.

Using this method, all pixels in the image were scanned and infected parts of the leaves were identified (yellow area in Figure 1b). Following this step, region of interest was defined to eliminate background. Healthy leaves in the canopy (Figure 1c) were identified using Eq. (2) with a manually selected threshold of 20, optimized during image processing for distinguishing the background from area of interest:

(2) $$I_{\left(i,j\right)}=\text{Healthy leaves pixels}(G_{\left(i,j\right)}>R_{\left(i,j\right)})\text{ and }(G_{\left(i,j\right)}-B_{\left(i,j\right)})>20)$$

Finally, total number of senesced and healthy pixels were computed and compared with ground reference data. In addition, the length of infected shoots was also calculated by selecting the two endpoints of the longest infected shoot. The coordinates of these two endpoints were obtained and the length of the infected shoot was computed. The three extracted features (maximum senesced shoot length, total senesced shoot leaf area, and ratio of senesced shoot leaf area with respect to healthy/green shoot leaf area) were compared with average senesced shoot length/tree, average proportion of shoot length blighted, and average tree disease severity rating based on wood age.

### Multispectral Image Acquisition, Image Processing, and Feature Extraction

Multispectral images were acquired at two scales: 7 m above ground level (AGL) and at 100 m AGL. An agricultural utility vehicle (AUV, John Deere Gator^TM^ XUV590i, John Deere, Moline, IL, United States) with a retractable mast (FM50-25, Floatagraph Technologies, Santa Barbara, CA, United States) and a camera mount was used to capture multispectral images from top-view (in 2016 and 2017, 7 m AGL) of the trees. A modified multispectral 3-band digital camera (Canon ELPH110 HS, Carlstadt, NJ, United States) with resolution of 4608 × 3456 and red channel replaced with NIR channel (680–800 nm) was mounted on the camera mount on the platform mast. AUV was driven along the rows at the speed of 0.5 m/s. The camera was operated in ambient light condition and was equipped with an SD card for data storage. In 2017, aerial multispectral images were collected using an unmanned octocopter (ARF OktoXL 6S12, HiSystems GmbH, Moormerland, Germany) integrated with a multispectral camera (Rededge, Micasense, Seattle, WA, United States) to capture red (R), green (G), blue (B), red edge (RE), and near infrared (NIR) band images. The imaging altitude was 100 m AGL. All the spectral reflectance data was radiometrically corrected using a reference panel (Spectralon Reflectance Target, Labsphere^®^, North Sutton, NH, United States) placed within the camera’s field of view.

**FIGURE 2:**
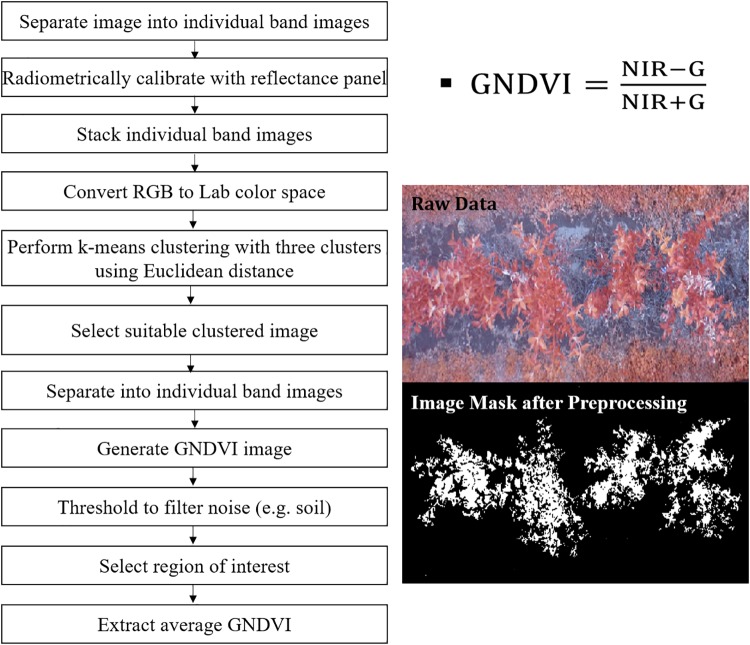
Data processing steps used to process AUV-based multispectral images.

Image processing and analysis were performed in Matlab^®^. For AUV-based multispectral images, five major steps of image processing were followed: (i) images were separated into individual bands and radiometrically corrected to compensate for incident light variation during the imaging; (ii) corresponding vegetation index images were extracted using Matlab^®^Image Processing Toolbox (Mathworks, Natick, MA, United States); (iii) the soil background and shadows were eliminated using a combination of k-means clustering and thresholding methods that discriminate regions of interest (trees) from the background; (iv) individual trees were segmented from the preprocessed image; and (v) the green normalized difference vegetation index (GNDVI) was extracted from segmented trees and the data was recorded by matching their ID. Figure 2 describes these steps in detail.

For the aerial images, the mosaic of NIR band was selected as the reference image to align images in other bands. Mosaics of red, green, blue, and red edge bands were geometrically corrected to match spatially with the NIR band. This process was performed using the “georeference tool” in QGIS software (QGIS Development Team, Graphic Information System, Open Source Geospatial Foundation Project^1^). With the same software, the five bands were merged into one color composite image following their conventional order: (1) red, (2) green, (3) blue, (4) red edge and (5) NIR, using the “Build Virtual Raster” command. In AutoCAD (Autodesk Inc., San Rafael, CA, United States), using the “Polyline” command, vector layers were created to isolate the area of each tree. For this purpose, a polygon surrounding the entire tree canopy area was developed. Later, the canopy area polygon was segmented into smaller fields of view (polygon) of 0.79 cm length, representing each tree. The sum and average tree GNDVI, normalized difference red edge index (NDRE), and normalized difference vegetation index (NDVI) were extracted using “Zonal Statistics” plugin in QGIS. The following equations describe the vegetation indices:

(3) $$GNDVI=\frac{NIR-G}{NIR+G}$$

(4) $$NDRE=\frac{NIR-RE}{NIR+RE}$$

(5) $$NDVI=\frac{NIR-R}{NIR+R}$$

where: NIR, G, RE, and R refer to digital number representing reflectance at near infrared, green, red-edge, and red bands. The NDVI (Rouse et al., 1973) and GNDVI (Gitelson and Merzlyak, 1998) are broadband greenness indices that represent overall canopy vigor or greenness; while NDRE (Gitelson and Merzlyak, 1994; Sims and Gamon, 2002) is a narrowband greenness that represents canopy stress response. In addition, NDRE also captures differences in foliage content and senescence, which could be useful in capturing disease symptoms. Vegetation index data were correlated to ground reference data (average disease severity rating based on wood age) during analysis.

### Hyperspectral Data Acquisition, Processing, and Feature Extraction

In addition to remote sensing data, proximal visible-near infrared (Vis-NIR) reflectance spectra using spectroradiometer (SVC HR-1024i, Spectra Vista Corporation, Poughkeepsie, NY, United States) was captured from the tree leaves under study (2016 and 2017). This hyperspectral system measures reflectance in the range 350–2,500 nm (overall 992 channels) with resolution of ≤3.5 at 700 nm, ≤9.5 at 1,500 nm, and ≤6.5 nm at 2,100 nm. The leaf clip with fiber optic channel connected to the equipment was used during data collection. Hyperspectral data was collected from two inoculated shoots from each tree (one young leaf on the shoot tip in the vicinity of the inoculated leaf and one mature leaf from new season’s growth). During analysis, in 2016, average disease rating was utilized; while in 2017, the disease rating for measured shoots was used.

Hyperspectral reflectance data captured using spectroradiometer was radiometrically corrected, normalized (Sankaran et al., 2011), and binned with 10 nm intervals. Two models, partial least square regression (PLSR) and quadratic kernel support vector machine (QSVM) were applied to classify spectra into four classes of 0, 1, 2, and 3 that represent disease rating (ground reference data), by separating the dataset into train and test datasets with 3:1 ratio after randomization. PLSR is a regression model that takes structures of both explanatory and independent variables into account. Variables are decomposed into latent structures in an iterative method. QSVM uses kernel to transfer data to a quadratic space and then defines a linear decision boundary. For two-class classification, 0 and 1 ratings and 2 and 3 ratings were combined. For three-class classification, only classes of 2 and 3 were combined. Overall classification accuracy was computed to assess performance of the class during four-class, three-class, and two-class classification accuracies.

Following hyperspectral data-based classification, binned reflectance data were utilized to generate normalized difference spectral indices (NDSIs) (Inoue et al., 2008), that represents every possible coupled combination of reflectance wavelengths with following equation:

(6) $$NDSI\left[i,j\right]=\frac{R_{i}-R_{j}}{R_{i}+R_{j}}$$

where *R* refers to reflectance data, and *i* and *j* refer to specific spectral bands. These spectral indices evaluate novel combinations of spectral bands, from which spectral ratios that were closely related to disease symptoms were selected. This would increase the accuracy of disease symptom assessment with few spectral bands rather than entire hyperspectral data.

**FIGURE 3:**
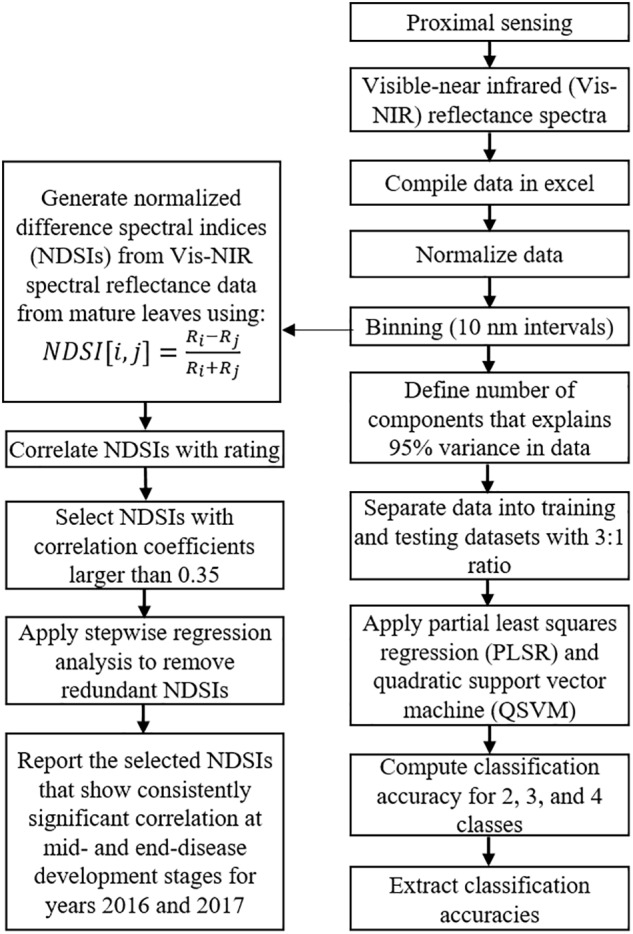
Data processing steps used to process hyperspectral reflectance data.

Normalized difference spectral indices were extracted from Vis-NIR spectral reflectance data of mature leaves from inoculated trees at mid- and complete-disease development stages for two consecutive years. Considering 214 spectral features after binning, 45,796 NDSIs were calculated for each spectrum, which were correlated with ground reference measurements for each tree. NDSIs correlation coefficients over 0.35 were selected for further analysis. To remove data redundancy from selected NDSIs, stepwise regression analysis was applied to each dataset. This method is a variable selection procedure for independent variables. It consists of a series of steps to evaluate each variable with a defined criterion in order to decide if it should be selected. In this study, only NDSIs that had the highest correlations were finally selected and redundant indices were removed. Figure 3 describes the data processing steps used for hyperspectral reflectance data analysis. All Pearson’s correlation analyses between extracted features and ground reference data were performed in R program (version 3.1.1, R Foundation for Statistical Computing, Vienna, Austria).

## Results

### Disease Rating and Stomatal Conductance

**FIGURE 4:**
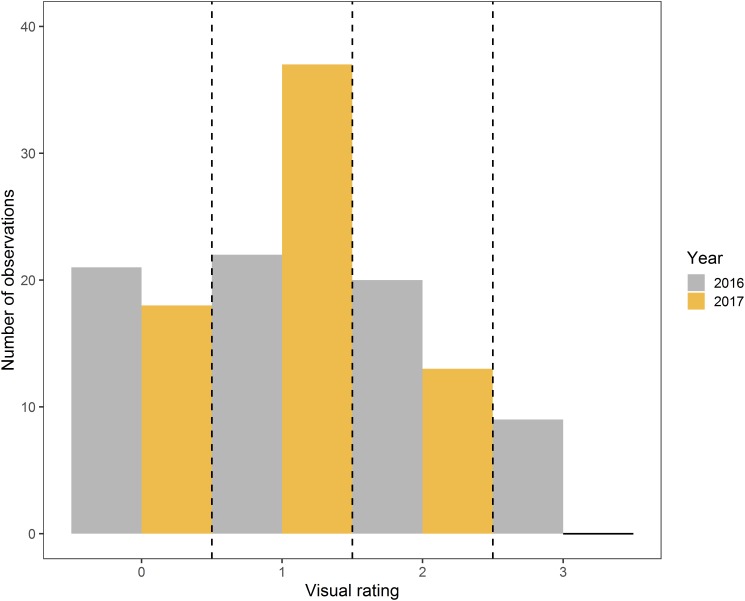
Distribution of samples in four disease severity rating classes based on the age of the wood infection in 2016 and 2017.

Proportion of shoot length blighted (Norelli et al., 2003; Khan et al., 2006; Durel et al., 2009) and the age of infected wood (Harshman et al., 2017) for fire blight phenotyping have been used in different studies. In this study, length of healthy tissue on inoculated shoots was measured right after inoculation and at the complete disease developmental stage. Disease severity was also rated according to age of wood infected on each inoculated shoot; the tree average was considered as actual disease severity rating. Ratings based on proportion of shoot length blighted were highly correlated with disease severity rating based on age of wood infected in 2016 (*r* = 0.96) and 2017 (*r* = 0.93). Therefore, for most parts of this study, disease severity based on the age of infected wood was used as a ground reference measure (Figure 4). While analyzing side-view RGB images, proportion of shoot length blighted was also considered, as it was more related to the image features.

Stomatal conductance measurements were collected from inoculated and healthy shoots in the experimental trees. How the bacteria travels through the plant tissue has yet to be fully determined; however, there is reasonable evidence that *E. amylovora* travels through intercellular spaces as described in the review paper (Billing, 2011). We anticipated that as this process of infection can affect water use efficiency, leaf stomatal conductance can be used to evaluate disease progression. Data were analyzed by computing the difference between stomatal conductance data from both healthy and inoculated shoots within the same tree (to normalize data for variety differences on the stomatal conductance) and correlating this normalized stomatal conductance difference with disease severity rating. Results showed a statistically significant correlation among these parameters (*r* = 0.51, *P* < 0.05) with an increase in normalized stomatal conductance values (representing higher differences between data from inoculated and healthy shoot leaves) associated with increased disease severity rating based on wood age.

### RGB Side Imaging

Three features were extracted from RGB side-images (2017): (1) maximum length of shoots with senesced leaves (pixels), (2) total area of senesced leaves (pixels), and (3) ratio of senesced to healthy leaf area. These three features were compared to actual lesion length, proportion of shoot length blighted, and disease severity rating based on wood age. The correlation coefficients were significant (*P* < 0.01) and are summarized in Table 1. In general, all RGB image features were correlated with ground reference data. Amongst these features, the strongest correlation coefficient of 0.51 was found between maximum length of shoot with senesced leaves as measured using RGB imaging and total lesion length. The direct relationship between these two measures could be the reason for high correlation.

**Table 1:** Correlation coefficient (*r*) between RGB image features and disease severity rating based on wood age in 2017.

Ground reference data	Total lesion length	Proportion of shoot length blighted	Disease severity rating based on wood age
Maximum length of shoot with senesced leaves	0.51	0.46	0.47
Total area of senesced leaves	0.45	0.39	0.42
Pixel ratio of senesced leaf area/healthy leaf area	0.41	0.36	0.38


### Multispectral Imaging

**FIGURE 5:**
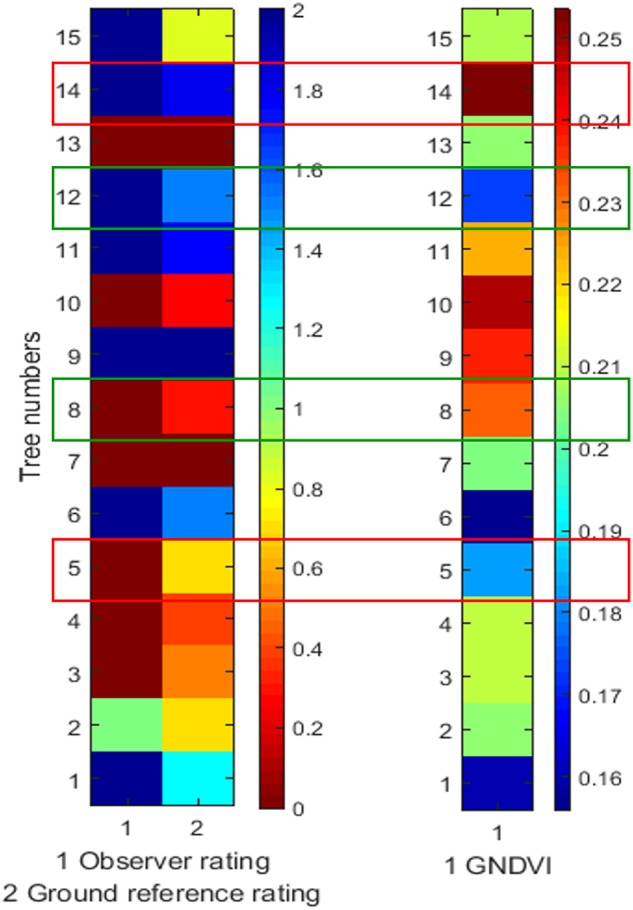
Color map showing disease rating and GNDVI data of representative 2016 diseased trees. Ground reference rating refers to disease rating; while observer rating refers to non-expert evaluator. Green box represents similar disease rating-GNDVI trend and red box represents dissimilar disease rating-GNDVI trend.

Multispectral imaging at multiple scales were evaluated to measure overall tree stress resulting from the inoculation. Although only inoculated shoots (limited in comparison to overall branches on the tree canopy) were expected to experience necrosis during disease development, we anticipated that this process will induce stress at the canopy level, which could be measured using remote sensing. During AUV-based multispectral imaging (2016 and 2017), in some cases, a similar trend between disease rating and GNDVI data was observed, even if the pattern was not consistent. Figure 5 shows the color map of a few sample trees from data collected in 2016, where ground reference rating refers to disease rating and observer rating refers to non-expert evaluator (S. Jarolmasjed). In 2016, a significant correlation between GNDVI and disease severity rating was observed (*r* = -0.38, *P* < 0.01), which was absent in 2017 (*r* = -0.22, *P* = 0.08, outliers were removed). Higher canopy volume in 2017 may have contributed to minor errors in tree segmentation or masking of symptoms, which could have led to these results.

**FIGURE 6:**
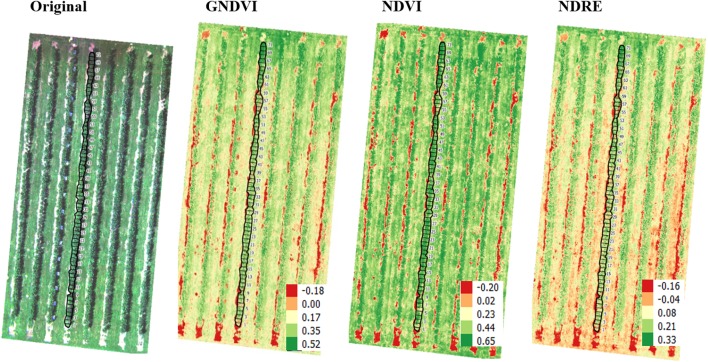
The original RGB, GNDVI, NDVI, and NDRE images of trees evaluated in 2017. The scales in GNDVI, NDVI, and NDRE represent the range of vegetation index data. The heterogeneity in tree canopy was a function of growth and disease status of each tree. The black vector superimposed on the original and vegetation index images represent segmentation of each tree.

In regard to UAV-based multispectral images (2017), the average and sum vegetation indices values (Figure 6) were extracted for individual segmented trees. These features were significantly (Table 2, *P* < 0.01) correlated to disease severity rating. Sensors used for phenotyping disease resistance should ideally be able to capture subtle differences in disease symptoms. It was interesting to note that although AUV-based multispectral images at higher resolution could not capture canopy health differences using vegetation indices, aerial images showed similar trend (similar to those using AUV-based multispectral images in 2016). Higher canopy vigor in 2017 (in comparison to 2016), combined with enhancement of image noise could have resulted in no correlation between AUV image-based GNDVI data and visual rating in 2017.

**Table 2:** Correlation coefficient (*r*) between UAV-based multispectral image features and disease severity rating based on wood age in 2017.

Statistic	Sum	Average
GNDVI	-0.38	-0.37
NDRE	-0.40	-0.37
NDVI	-0.35	-0.37


### Hyperspectral Spectrometry

**FIGURE 7:**
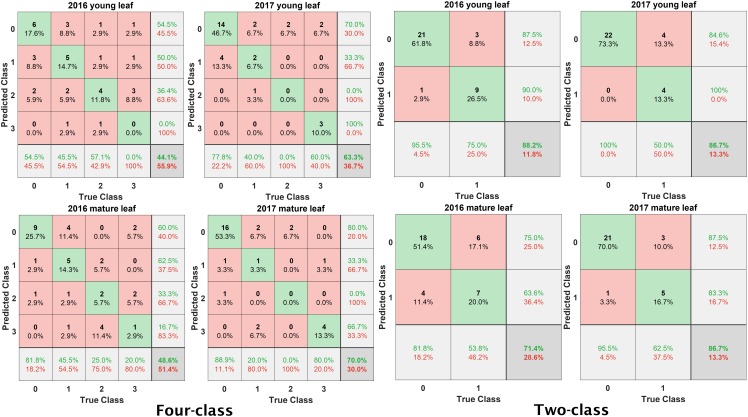
Confusion matrices of classification accuracies computed using quadratic support vector machine. The two classes represent 0 and 1 rating as class 0 and 2 and 3 rating as class 1; while four classes represent rating 0, 1, 2, and 3 as four classes.

The overall classification accuracies with PLSR and QSVM are summarized in Table 3. The purpose of the classification was to observe the Vis-NIR spectral reflectance pattern difference between different disease ratings. Results indicated that spectra are capable of delineating fire blight disease rating in shoots. In general, classification accuracies were higher in 2017 than 2016, which could be resulting from differences between disease developments in both years. In general, the classification of two classes was more accurate than four classes. Observing the confusion matrix (Figure 7), it was found that the classification accuracies with four classes were often lower because 0 and 1 ratings were often misclassified as 1 and 0 ratings, respectively; while 2 and 3 ratings were misclassified as 3 and 2 ratings, respectively.

**Table 3:** Overall classification accuracies computed using two models partial least square regression (PLSR) and quadratic support vector machine (QSVM).

		Overall classification accuracy (%)					

Year	Classifier	Four classes (rating: 0, 1, 2, 3)		Three classes (rating: 0, 1, 2–3)		Two classes (rating: 0–1, 2–3)	
				
		Young leaf	Mature leaf	Young leaf	Mature leaf	Young leaf	Mature leaf
2016	PLSR	62	46	47	54	91	74
	QSVM	44	49	56	49	88	71
2017	PLSR	60	63	50	63	93	90
	QSVM	63	70	70	70	87	87


**FIGURE 8:**
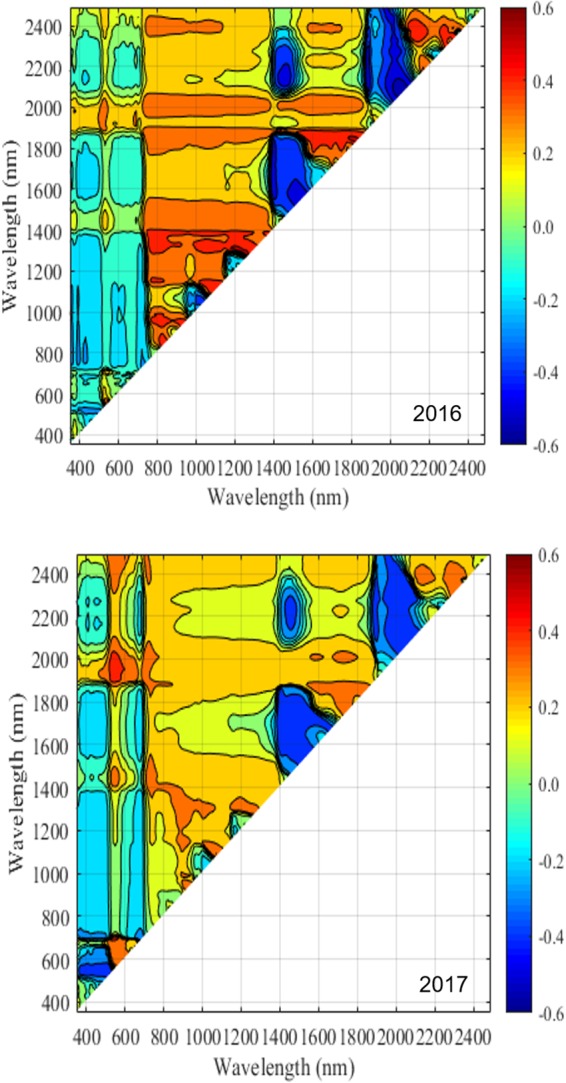
Correlation between NDSIs generated using hyperspectral data collected during the mid-season and disease rating in 2016 and 2017. The color scale represents Pearson’s correlation coefficients (*r*).

In regard to the NDSI selection, mature leaf spectra were used for analysis, as their spectral pattern was considered to be more stable during the season and disease development. Figure 8, 9 show the distribution of correlation coefficient between NDSIs generated with the entire spectra and disease rating using mid and end of (disease development) season datasets for 2016 and 2017. One interesting observation from this data is the consistency in the relationship (correlation) pattern across two seasons, at both mid-season and end of disease development period. This indicates development of fire blight infection may progress in a predicted manner. Moreover, stronger correlations between NDSIs and disease rating were found in end of season than mid-season, which confirms the development of symptoms at the end of disease development phase. Using the raw dataset of NDSI, stepwise regression was applied to select NDSIs that were significantly correlated with disease rating within a season (excluding visible bands). The final set of selected NDSIs are reported in Table 4. All NDSIs were significantly correlated with the visual ratings. Figure 10 shows the boxplot of representative NDSIs.

**FIGURE 9:**
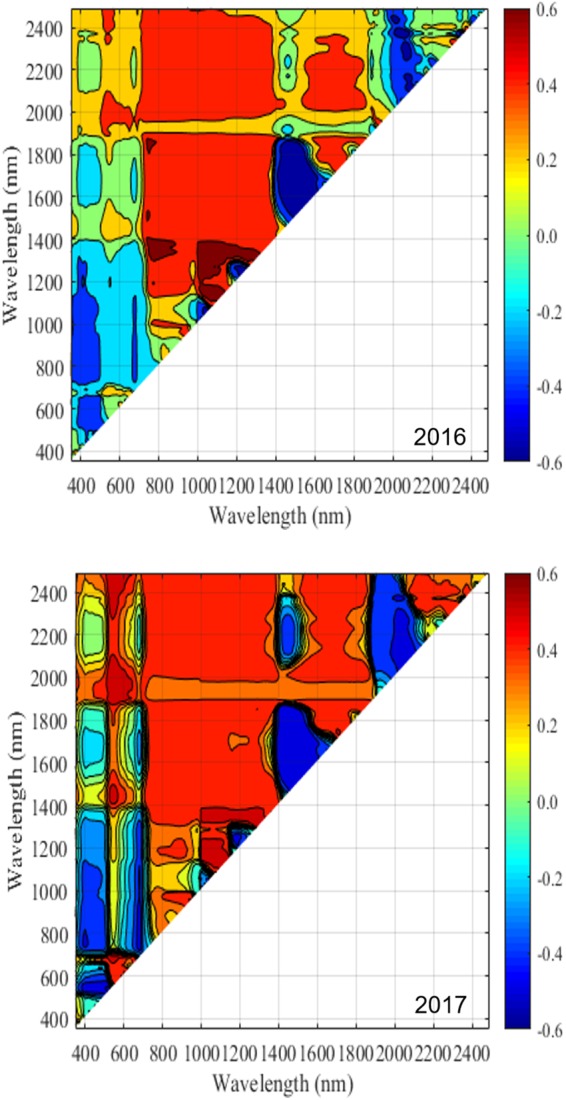
Correlation between NDSIs generated using hyperspectral data collected during the end of disease development and disease rating in 2016 and 2017. The color scale represents Pearson’s correlation coefficients (*r*).

**Table 4:** Selected wavelength combinations used in normalized different spectral index (NDSI) resulting from feature selection from 2016 and 2017 datasets.

		2016		2017	
			
Selected NDSI from year	Wavelength (nm)	Mid-season	End-season	Mid-season	End-season
2016	1170, 1320	0.47***	0.64***	0.36***	0.41***
	1420, 1880	0.53***	0.42***	0.29***	0.42***
	1520, 1620	-0.42***	-0.56***	-0.36***	-0.48***
	1560, 1570	-0.46***	-0.58***	-0.33***	-0.48***
	2040, 2240	-0.40***	-0.37***	-0.34***	-0.41***
2017	710, 2020	0.21*	0.46***	0.35***	0.46***
	1030, 1130	0.39***	0.61***	0.36***	0.50***
	2050, 2110	-0.53***	-0.36***	-0.37***	-0.44***
	2070, 2080	-0.55***	-0.31***	-0.38***	-0.45***
	2090, 2100	-0.51***	-0.46***	-0.36***	-0.45***
	2330, 2340	0.47***	0.38***	0.36***	0.47***


*The mid- and end-season refer to middle and end of disease development period. The correlation coefficients (*r*) are between NDSIs and disease severity rating by wood age using selected wavelength combinations. Statistical significance: ^∗^*P < 0.05*; ^∗∗∗^*P < 0.0001*; ns, not significant.*

**FIGURE 10:**
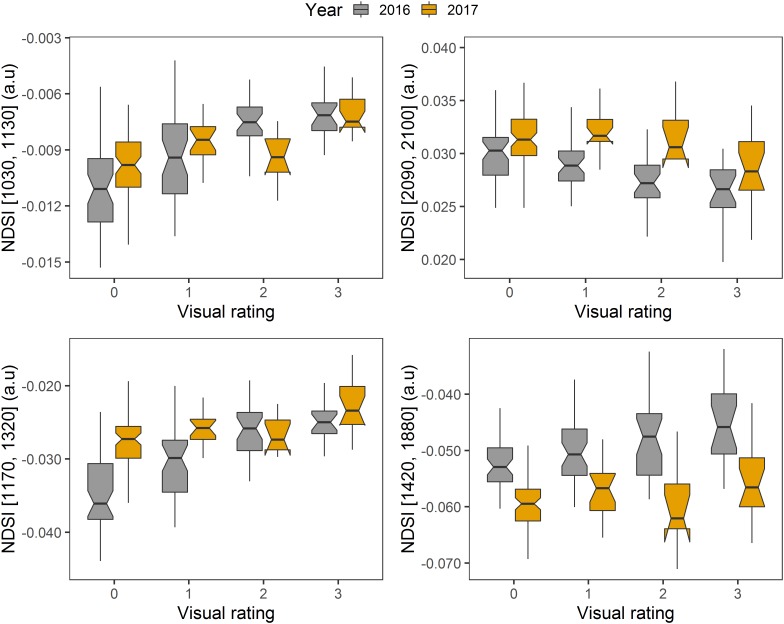
Boxplots of selected NDSIs generated using 2016 and 2017 end of disease development phase datasets. The visual rating refers to disease severity rating by wood age.

## Discussion

Apple breeders need to evaluate the performance of their germplasm under different conditions in order to select varieties that are more resistant to disease/other conditions, which is also an important aspect of integrated pest management (Lespinasse and Aldwinckle, 2000). In this study, WSU apple germplasms were inoculated with the bacteria causing fire blight (*E. amylovora*), a potentially devastating pathogen. Fire blight causes significant production loss for the commercial fruit industry worldwide, and consequently, resistant cultivars are sought to save crops from its devastating effects. Typically, phenotyping susceptibility to fire blight shoot infection is performed by estimating incidence under natural infection pressure or measuring shoot lesion length after artificial inoculation. These options are subjective and labor intensive (Lespinasse and Aldwinckle, 2000). For these reasons, high-throughput phenotyping techniques offer standardization in the process of disease rating in an accurate and rapid manner. In this study, multiple sensing systems at ranging complexity (RGB, multispectral, and hyperspectral sensing systems) were evaluated to select the most effective and accurate method for disease severity evaluation in apple. The benefits and limitations of each method is reported in Supplementary Table S2.

In regard to RGB imaging, a canopy trait such as senescence can be easily captured (Ahmad and Reid, 1996; Laliberte et al., 2007). RGB images were explored to estimate the senescence leaf area from captured data and the extracted features showed significant correlation with disease rating. One major limitation in the throughput of this method was placement of white background. Accuracy and throughput of such evaluation can be further enhanced with the use of an automated phenotyping system to cover the canopy for controlled lighting and imaging conditions, in addition to accurate estimation of distance between the canopy and the sensor using 3D camera or other time-of-flight sensors. Moreover, image processing protocol utilized in this study could not delineate minor differences between branches/shoots and senesced leaves, although noise from other tissues could be eliminated. Even if this noise was minimal, integrating image processing techniques such as Hough transform and machine learning algorithms can potentially increase accuracy of this technique. Nevertheless, other stress conditions influencing leaf color will affect the accuracy of this method.

Multispectral cameras were also integrated with multiple platforms (AUV, UAV) to capture images in order to phenotype disease rating and assess fire blight symptoms. The vegetation indices extracted from multispectral images acquired from AUV (GNDVI, 2016) and UAV (GNDVI, NDRE, NDVI; 2017) showed weak (yet significant) correlation with disease rating. In 2017, correlation between GNDVI and disease severity rating was absent, which could be associated with segmentation issues during image processing as reported in Laliberte and Rango (2009). Overlapping shoots could not be detected from the images in some cases (especially when the canopy vigor was high) as the trees were planted at high density.

The significant correlation between vegetation indices extracted from remote sensing data with disease severity rating showed the capability of vegetation indices to detect differences in the canopy reflectance between symptomless and susceptible trees, although with low sensitivity (Gröll et al., 2007). The observed result in our study were in contrast with the study reported in Naidu et al. (2009), where significant differences between the vegetation indices of non-infected and infected plants with leaf roll-associated virus-3 in grapevine were not found. On the other hand, Mahlein et al. (2013) achieved an accuracy of 80% while classifying Cercospora leaf spot infected and healthy leaves using NDVI in sugarbeet. In the same crop, a correlation coefficient *r* of -0.89 was found between NDVI and diseased leaf severity (Cercospora leaf spot, powdery mildew; Mahlein et al., 2010). The efficacy in the detection of plant diseases using vegetation index depends on the crop, pathogen, and symptoms. Although AUV and UAV-based multispectral images may provide information of canopy health, the limited number of spectral bands will limit its application in disease-specific evaluation, which can be better achieved with data capture in broader visible-near infrared spectral reflectance range in comparison to using vegetation indices (Montesinos-López et al., 2017). The remote sensing technique can be used for scouting for fire blight disease incidence in production systems, which can be followed by pathological evaluation of samples.

At times, vegetation indices do not provide relationship with crop stress (Eitel et al., 2008; Jarolmasjed et al., 2018). NDVI has also been reported to record plant responses to water stress, and are indicative of chlorophyll and yield (Ceccato et al., 2002; Gitelson et al., 2003; Eitel et al., 2008). Moreover, the specific absorption coefficient of chlorophyll in the red channel is high, and when combined with the low depth of light penetration into the leaf, the index can create saturation. For a similar reason, NDVI cannot capture stress responses from a canopy with higher leaf area index (Gitelson et al., 2003). In this regard, hyperspectral sensing offers a more specific evaluation of certain disease.

Visible-near infrared spectrometry was used to capture the spectral reflectance of the leaves and create a set of novel vegetation indices (NDSIs) representing fire blight disease symptom progression. Such indices were created utilizing the high-spectral resolution visible-near infrared reflectance data (Inoue et al., 2008). Other feature extraction methods reported in literature (Sun et al., 2017; Sun and Du, 2018) can also be explored for hyperspectral data analysis. In this study, the highest correlating NDSIs to visual rating were reported as spectral bands capable of defining fire blight disease in apple trees. The selected wavelengths for NDSIs ranged from 710 to 2340 nm. Among different wavelengths, the red edge band (696–704 nm) reported to contain a high amount of information in regard to chlorophyll content and vegetation stress (Mohd Shafr and Hamdan, 2009) was also present. In a lettuce maturity detection study (Brach et al., 1982), the ratio of 1,170 nm to 1,110 nm provided in morphogenetic differentiation of the tissue. The 1,170 nm was also selected as one of the wavelength features in this work. Few wavelengths reported in literature that could discriminate wolfberries (Yin et al., 2017) such as 1,130, 1,160, 1,300, 1,328, and 1,423 nm were also similar to the wavelengths during NDSI selection in the current study. The selected NDSIs found in this research were consistently highly correlated across seasons, and showed a prospective for early fire blight disease detection that can be useful in precision agriculture applications. Thus, imaging and spectrometric techniques have the potential to be used as phenotyping tool that are dependent on the crop and disease conditions. In future, multispectral imaging with customized spectral band combinations can be used for fire blight resistant evaluation.

## Conclusion

In summary, the RGB and multispectral imaging sensors offered low to moderate accuracy in detection of disease severity based on image features representing senescence and vegetation indices extracted from the images, respectively. The absence of higher spectral resolution in the process of disease severity evaluation in breeding programs can limit the application of these sensing systems. In addition, presence of other stress conditions such as heat stress and/or other diseases, may also limit the potential use of these techniques. In this regard, hyperspectral sensing system can capture disease-specific responses that can be useful for disease severity evaluations. The normalized difference spectral ratios derived from hyperspectral data were found to show moderate to high accuracies in disease severity evaluation, which were consistent between two seasons. Thus, these specific spectral bands could be used for evaluating fire blight disease severity in apple breeding programs. In addition, these indices also showed potential to be used as early disease detection tools that could assist in timely crop management in production systems.

## Author Contributions

SJ, SS, and KE: conceptualization. SJ, SK, and KE: data collection. YS and SS: data processing and analysis – RGB images. JQ and SS: data processing and analysis – UAV images. SJ and SS: data processing and analysis – proximal multispectral images, hyperspectral data, and writing – original draft preparation. SS and KE: resources, supervision, project administration, and funding acquisition. SK, KE, and AM: writing – review and editing. SJ, AM, JQ, and SS: visualization.

## Conflict of Interest Statement

The authors declare that the research was conducted in the absence of any commercial or financial relationships that could be construed as a potential conflict of interest.

## Footnotes

1 http://qgis.osgeo.org
