# Supplementary material for: High-Throughput Phenotyping of Fire Blight Disease Symptoms Using Sensing Techniques in Apple
Source: Front Plant Sci. 2019 May 10;10:576. doi: 10.3389/fpls.2019.00576 (PMC6523796; doi:10.3389/fpls.2019.00576)
Supplement: Supplementary file 1 [file Data_Sheet_1.docx]

Supplementary Material

**High-Throughput Phenotyping of Fire Blight Disease Symptoms using Sensing Techniques in Apple**

Sanaz Jarolmasjed^1†^, Sindhuja Sankaran^1*†^, Afef Marzougui^1^, Sarah Kostick^2^, Yongsheng Si^3^, Juan José Quirós Vargas^1^, Kate Evans^2^

^1^Department of Biological Systems Engineering, Washington State University, PO Box 646120, Pullman, WA 99164, USA

^2^Tree Fruit Research and Extension Center, Washington State University, 1100 N Western Ave Wenatchee, WA 98801, USA

^3^College of Information Science and Technology, Agriculture University of HeBei, Baoding, HeHei, China

***Correspondence:** Sindhuja Sankaran, [sindhuja.sankaran@wsu.edu](mailto:sindhuja.sankaran@wsu.edu)

^†^Equal contributing author

# Supplementary Figures and Tables

## Supplementary Figures

**
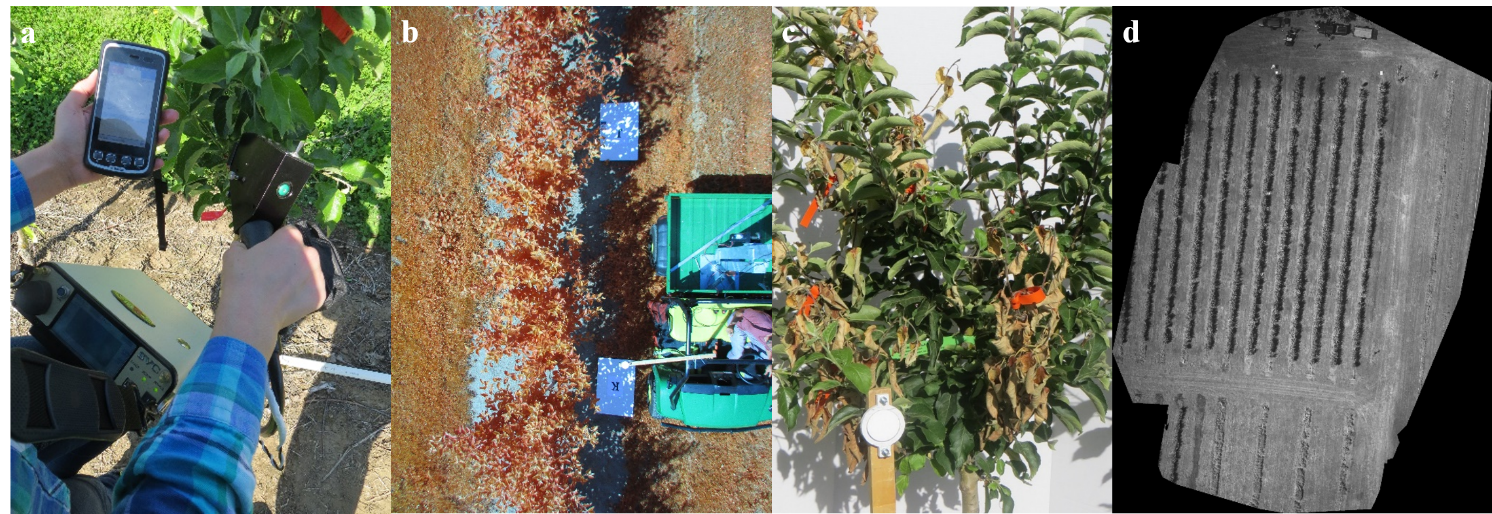
**

**Supplementary Figure S1.** Sensor-based data collection: Proximal sensing with: (a) Visible-near infrared spectroradiometer; (b) AUV-based multispectral imaging (top-view); (c) AUV-based RGB imaging (side-view); and (d) UAV-based aerial multispectral imaging (stitched near infrared band). Images are not to scale.

## Supplementary Tables

**Supplementary Table 1.** Summary of type of data collected, analyzed, and brief results from this study.

| Evaluation method/Sensor | Proximity | Data analysis method | Feature(s) extracted | Year | Brief Results |
| --- | --- | --- | --- | --- | --- |
| Disease severity rating (visual/manual measurement) | Proximal | - | Age of wood infected; proportion of shoot length blighted | 2016, 2017 | Not applicable |
| Portable Photosynthesis system (LI-6800) | Proximal | - | Stomatal conductance | 2017 | *r*=0.51, P<0.05 |
| RGB Camera | 1.5 m (side-view) | Image thresholding using R, G and B bands to distinguish healthy and necrotic leaves; regions of interest were defined; thresholding was performed to separate background; features were extracted. | Maximum senesced shoot length, total senesced shoot leaf area, ratio of senesced shoot leaf area with respect to healthy/green shoot leaf area | 2017 | *r*=0.36-0.51, P<0.01 |
| Multispectral Camera (NIR, G, B) | 7 m | Radiometric correction using reference panel; GNDVI images were extracted; soil and shadow were eliminated using k-means clustering; regions of interest were defined; average GNDVI was extracted. | GNDVI | 2016, 2017 | \|*r\|*=0.38, P<0.01 (2016); \|*r\|*=0.22, P=0.08 (2017) |
| Multispectral (R, G, B, RE, NIR) | 100 m | Data were radiometrically corrected; multiband images were aligned with NIR mosaic; color composite image was generated; each tree area was defined through polygon generation; sum and average vegetation index values were extracted. | GNDVI, NDVI, NDRE | 2017 | \|*r\|*=0.35-0.40, P<0.01 |
| Hyperspectral reflectance data (350-2500 nm) | Proximal | Data was radiometrically corrected, normalized, and binned (10 nm); classification into 4, 3, and 2 classes was performed using QSVM and PLSR; NDSIs were generated; correlation coefficient thresholding was performed to select key NDSIs, redundant features were removed using stepwise regression analysis for selecting important NDSIs. | 11 NDSIs (710-2340 nm) | 2016, 2017 | 71-93% classification accuracy (two-class); \|*r\|*=0.21-0.61, P<0.05 |

**Supplementary Table 2.** Benefits and limitations of sensing techniques

| Sensor | Proximity | Benefits | Limitations |
| --- | --- | --- | --- |
| Portable Photosynthesis system (LI-6800) | Proximal | - Measure of physiological status | - Low throughput - Time consuming - Reference measure for each genotype can be different |
| RGB Camera | 1.5 m (side-view) | - Low cost sensor - Moderate accuracy - Measure of distance between the sensor and tree and use of other techniques can enhance accuracy | - Some noise contributed from shoots - Limited to visible symptoms |
| Multispectral (G, B, NIR) imaging from top | 7 m & 100 m (top-view) | - Low to moderate cost sensor - Moderate accuracy - High-throughput phenotypic assessment is possible | - Other disease/stress can result in similar response - Limited shoots that were inoculated with pathogen is hard to detect |
| Hyperspectral reflectance data (350-2500 nm) | Proximal | - High cost sensor - Moderate to high accuracy - Can be disease specific - Cost can be reduced with customized multiband sensor development with selected spectral bands | - Hyperspectral data processing and analysis can be complex - Selected spectral bands need to be validated |
